# Physical activity and sedentary behavior profiles among prisoners in an open prison: differences in psychological well-being?

**DOI:** 10.1186/s12889-025-24345-0

**Published:** 2025-09-01

**Authors:** Christian Kraft, Bernd Gröben, Pamela Wicker

**Affiliations:** Department of Sports Science, Bielefeld University, Universitaetsstr. 25, 33615 Bielefeld, Germany

**Keywords:** Mental health, Well-being, Physical activity, Prison, Incarcerated, Sedentary lifestyle

## Abstract

**Background:**

Incarcerated individuals face elevated risks for both physical and mental health issues. While structured physical activity programs have shown health benefits, little is known about how general patterns of physical activity and sedentary behavior, including screen-based media use, unfold in everyday prison life. This study aimed to identify distinct behavioral profiles among prisoners and explore whether these groups differ in psychological well-being indicators.

**Methods:**

A paper-pencil survey was conducted in a German open prison for young male offenders. Self-reported data were collected on daily physical activity at different intensity levels (vigorous, moderate, and walking as a proxy for light intensity), sedentary time (sitting, television), and psychological well-being indicators. A two-step cluster analysis was performed to identify behavioral subgroups. Group comparisons on psychological measures were conducted using analysis of variances and Welch tests with post-hoc comparisons.

**Results:**

Three behavioral profiles emerged: (1) high media consumption with low-intensity activity, (2) highly active with low sedentary behavior, and (3) low activity with low media time. Clusters differed significantly in all physical and sedentary behavior indicators. The weekly metabolic equivalent of task minutes also varied significantly across the sample. On average, prisoners reported 5,410 metabolic equivalent of task minutes per week, substantially exceeding the minimum levels recommended by the World Health Organization. Only positive affect differed significantly across psychological variables, with higher scores in the highly active, low sedentary group compared to the high media, low-intensity group.

**Conclusions:**

The findings demonstrate substantial variation in daily activity and media patterns among prisoners in open correctional settings. Despite clear behavioral differences, psychological well-being was comparable between groups. This finding suggests that movement and media routines should be considered when promoting prison health, yet they are not coupled with well-being differences under restrictive conditions. A cluster-based approach offers a useful basis for developing tailored interventions for different physical activity profiles among prisoners.

**Supplementary Information:**

The online version contains supplementary material available at 10.1186/s12889-025-24345-0.

## Background

Prisons pose significant risks to both physical and mental health [1, 2]; since, incarcerated individuals face high levels of psychological distress and have limited access to health-promoting behaviors [1, 2]. As imprisonment is typically temporary, prisoner health also impacts the broader public health after release [1, 2]. Within the framework of equivalence of care, prisoners are entitled to the same standard of healthcare as the general population [1, 3]. Many inmates come from disadvantaged backgrounds and may experience regular healthcare for the first time during incarceration [1, 3]. This includes access to adequate physical activity (PA) and sports participation [1, 3]. However, because of its spatial restrictions and fixed routines, the prison context often limits movement and can promote sedentary behavior, such as prolonged sitting or watching television (TV) [1, 4].

PA and sedentary behavior are both key behavioral factors affecting health. In the general population, insufficient PA and high sedentary behavior are linked to poorer physical and mental health outcomes [5–8]. While the guidelines of the World Health Organization (WHO) offer benchmarks for healthy activity [9], little is known about how open prison routines shape activity and sedentary behavior. These recommendations emphasize that greater health benefits - particularly mental, cardiovascular, and metabolic health outcomes - can be achieved through longer and/or higher intensity activity [9]. Accordingly, it is essential to assess PA across intensity levels, such as vigorous and moderate activity, as well as walking (used here as a proxy for low-intensity PA) to understand how prison settings may support or hinder health-promoting behavior. This is especially relevant in open prisons where inmates are permitted to leave the facility for work, education, or family visits, allowing prisoners more autonomy in shaping their daily routines, including opportunities for PA and other leisure activities.

Previous studies in the prison context have primarily examined structured sports or intervention programs (e.g., yoga, strength training, team sports) and their effects on physiological and psychological outcomes [10–13]. While these studies provide valuable insights, they do not reflect the daily unstructured activity and sedentary behavior patterns of prisoners. One of the most prominent sedentary activities in prison is watching TV, yet its psychological implications remain underexplored. Existing evidence has linked prolonged TV use to poorer mental health and lower psychological well-being [14, 15]; although, in carceral settings, it may serve as a coping mechanism or as a source of perceived autonomy [16]. More broadly, sedentary behavior, regardless of screen use, is recognized as an independent risk factor for adverse physical and mental health outcomes, including increased mortality risk [14, 15]. Some studies even suggest that excessive sedentary time can cause irreversible harm if prolonged beyond a certain threshold [14]. Notably, Mannocci et al. [17, 18] assessed general PA levels using metabolic equivalent of task (MET) minutes and found associations with quality of life among Italian prisoners. However, they neither distinguished between activity intensities nor identified distinct behavioral profiles. This leaves a knowledge gap in understanding how various intensities of PA and different forms of sedentary behavior, particularly screen-based media use, interact in daily prison life, especially in open prisons where inmates experience greater autonomy.

Given its recognized relevance in correctional contexts, this study also considers prisoners’ psychological well-being (PWB) of prisoners due to its recognized importance in the prison context [1–3]. PWB is considered the broadest and most comprehensive construct of well-being [19]. It is a multicomponent construct [20–22], encompassing subjective well-being (life satisfaction and evaluative judgment), hedonic well-being (positive affect and pleasure), eudaimonic well-being (meaning, personal growth, autonomy), and psychological ill-being (e.g., anxiety, depression) [10, 19]. Ryff and Keyes [23] conceptualize eudaimonic well-being through six core dimensions: autonomy, environmental mastery, personal growth, positive relations with others, purpose in life, and self-acceptance [24–26]. This study adopts a broad and inclusive perspective, integrating all four components in order to capture different facets of mental functioning behind bars.

Despite existing insights, several research gaps remain: (1) habitual PA and sedentary behavior in open correctional facilities are still understudied; (2) PA intensities (e.g., vigorous, moderate, and walking as a proxy for light intensity) are rarely differentiated, even though they relate to distinct health outcomes; (3) watching TV has received little attention despite its central role in prison life; (4) integrated behavioral profiles combining activity and sedentary patterns have not been explored; and (5) PWB is often assessed unidimensionally, with limited use of multidimensional frameworks.

Therefore, this study aims to identify distinct behavioral profiles of PA and sedentary behavior, including intensity levels and media use among prisoners in an open correctional facility. Following, PWB indicators are explored to examine whether variation in behavior coincides with differences in mental well-being. Addressing these research gaps, this study advances the following research questions (RQ): (RQ1) What typical patterns of physical activity and sedentary behavior, including intensity levels and screen-based behavior, can be identified among prisoners in an open prison?; and (RQ2) Do these behavioral profiles differ in terms of psychological well-being indicators?

By answering these questions, the study contributes to a behavioral and context-sensitive understanding of prisoner health. Rather than examining isolated behaviors or structured interventions, it offers a data-driven perspective on how PA and sedentary routines unfold in daily open prison life. These insights may help to inform profile-based, low-threshold health promotion strategies tailored to prisoner subgroups within institutions and during post-release reintegration.

## Methods

### Data collection and ethics

Data were collected in an open prison located in North Rhine-Westphalia, Germany, which specializes in young male offenders, typically between the ages of 18 and 25. The prison facility had a capacity of 232 inmates, but at the time of data collection, only approximately 100 individuals were incarcerated. Given the small total population of incarcerated individuals, data were collected in two waves to increase the sample size and to benefit from the high turnover in the investigated prison. The first data collection took place between January and March 2024 and the second wave between February and April 2025. This study utilized a cross-sectional design and collected data through paper-pencil surveys. Prisoners were recruited either by the corresponding author or by prison staff. Those who expressed interest in participating received envelopes containing the questionnaire. Participants were informed that their participation was voluntary and would not face any disadvantages if they chose to withdraw from the study. To ensure privacy, prisoners were allowed to complete the questionnaires in their cells. Once completed, the sealed envelopes were gathered either by the corresponding author or by prison staff.

In both waves, a total of 160 questionnaires were distributed, and 145 completed questionnaires were returned. After data cleaning, 17 questionnaires were excluded due to missing values on key variables or logical flaws (e.g., time of imprisonment longer than prison sentence, total daily activity hours exceeding 16 h). Consequently, 128 questionnaires were included in the final analysis. The study received ethical approval from the Bielefeld University Ethics Review Board (approval number: EUB-2023-169-S) and was conducted in accordance with the principles outlined in the Declaration of Helsinki.

### Questionnaire and variables

Table 1 displays the variables and their descriptions used in this study. The questionnaire was specifically developed for the present study to capture PA, sedentary behavior, PWB, and incarceration-related factors. Established and validated instruments were incorporated into the questionnaire to ensure conceptual and measurement rigor. The questionnaire opened with an introduction informing participants about the survey’s topic and their anonymity. PA and sedentary behavior were measured through the International Physical Activity Questionnaire (IPAQ) [27]. Subsequently, PWB was assessed through a multidimensional set of validated instruments. First, life satisfaction [28] was assessed, followed by the Brief Symptom Inventory (BSI-18) [29], Ryff’s psychological well-being scale [30], and the positive and negative affect scale (PANAS) [31]. Lastly, the questionnaire assessed sociodemographic variables and incarceration-specific questions. An English version of the complete questionnaire is provided as Supplementary Material 1.

**Table 1:** Variables overview and descriptive statistics (*n* = 128)

Variable	Description and Codes	Mean	SD
Daily vigorous PA	Minutes of vigorous physical activity per day	42.24	41.64
Daily moderate PA	Minutes of moderate physical activity per day	58.18	54.52
Daily walking PA	Minutes spent walking per day	61.29	65.80
Daily sitting time	Total hours spent sitting per week while being awake	5.34	3.40
Daily TV time	Total hours spent watching TV per week	3.24	2.55
Weekly MET	Weekly MET score from the IPAQ, representing total energy expenditure in MET minutes per week	5410.13	3847.76
Life satisfaction	On the whole, how satisfied are you currently with your life? (0 = not at all satisfied; 10 = totally satisfied)	5.81	2.77
GSI	Mean index of the three (depression, anxiety, and somatization) subscales (0 = no distress; 4 = maximum distress); Table 2	0.52	0.53
Eudaimonic well-being	Mean index of eudaimonic well-being; consisting of six subscales (1 = low eudaimonic well-being; 7 = high eudaimonic well-being); Table 3	4.66	0.77
Positive affect	Mean index of ten items reflecting the positive effect items of the PANAS (1 = low positive affect; 5 = high positive affect); Table 4	3.00	0.77
Negative affect	Mean index of ten items reflecting the negative effect items of the PANAS (1 = low negative affect; 5 = high negative affect); Table 4	2.06	0.74
Age	Age in years	21.73	2.08
BMI	Weight (kg)/[Height (m^2^)]	25.57	4.18
Total prison sentence	Total sentence length (in months)	26.33	16.31
Prison sentence served	Total number of months of the current prison sentence already served (in months)	9.80	10.93

### Physical activity and sedentary behavior

PA was assessed using the IPAQ short-form [27], which was adapted to the prison context to reflect participants’ activity levels accurately. The IPAQ is a widely used self-report instrument developed for international surveillance of PA and is designed to assess activity levels over the previous seven days [32, 33]. The questionnaire captures PA across different intensity levels, including vigorous- and moderate-intensity activities, as well as walking as a proxy for low-intensity [27]. In the present study, the German version of the IPAQ short-form was used [34]. Data cleaning followed the guidance of the IPAQ scoring protocol [32]. The IPAQ short-form measures total physical activity without distinguishing between voluntary activities (e.g., leisure-time, sports, or exercise) and non-voluntary activities (e.g., routine-based or practical tasks, such as walking to assigned work duties). Consequently, the IPAQ short-form captures overall levels but not the underlying motivational context.

Intensity levels were defined according to the IPAQ scoring protocol and item phrasing (Supplementary Material 1) [27, 34]. Vigorous physical activity was characterized as activities requiring hard physical effort and causing substantially increased breathing, e.g., running or heavy lifting. Moderate physical activity was defined as involving moderate physical effort with a slight increase in breathing, including activities like cycling at a regular pace or strength training. This study used walking as a proxy for low-intensity activity. In line with the IPAQ protocol [32], all reported activities had to be performed for a minimum of 10 min per bout data were screened for completeness and plausibility, and extreme values were handled in accordance with the IPAQ scoring protocol [32].

To quantify participants’ activity levels, weekly MET minutes and the average daily minutes of activity across different intensity categories were calculated. Based on the IPAQ scoring protocol, standard MET values were assigned: 8.0 METs for vigorous activity, 4.0 METs for moderate activity, and 3.3 METs for walking [32]. Additionally, both total sitting time and time spent watching TV were assessed to gain a more comprehensive understanding of sedentary behavior. This inclusion was based on prior research indicating that prolonged bouts of watching TV, as a form of sedentary behavior, are particularly detrimental to mental health outcomes [14, 15]. Consequently, the average daily number of hours spent sitting and watching TV was calculated for the present study.

### Life satisfaction

The life satisfaction of prisoners was assessed using a single-item measure derived from the German Socio-Economic Panel [28], allowing for direct comparisons with the general population, which offers valuable context for understanding the broader implications of imprisonment on life satisfaction. Participants responded to the question “On the whole, how satisfied are you currently with your life?” using an 11-point Likert scale, ranging from 0 (not at all satisfied) to 10 (completely satisfied). This measure has been used in psychological and socio-economic research [35, 36], demonstrating high validity and reliability as an indicator of overall subjective well-being [35, 37].

### Brief symptom inventory

The BSI-18 is the short form of the Symptom-Checklist-90 [29, 38], and is used to measure psychological distress experienced in the most recent seven days. The BSI-18 consists of 18 items representing somatization, depression, and anxiety components with a scoring range from 0 (not at all) to 4 (very strong). The present study used the validated German version of the BSI-18 [39]. The resulting global severity index (GSI) was calculated by averaging these 18 items. The Cronbach’s α value of 0.888 indicated that the scale demonstrated very good reliability [40].

**Table 2:** Brief-symptom-inventory-18

Items (0 = not at all; 4 = very strong)	Mean	SD	Cronbach’s α
Depression (Index)	0.65	0.69	
Feeling no interest in things	0.67	0.80	
Feeling lonely	1.11	1.18	
Feeling blue	0.35	0.78	
Feelings of worthlessness	0.77	1.21	
Feeling hopeless about the future	0.79	1.14	
Thoughts of ending your life	0.20	0.58	
Anxiety (Index)	0.59	0.67	
Nervousness or shakiness inside	0.55	0.83	
Feeling tense or keyed up	1.20	1.15	
Suddenly scared for no reason	0.27	0.84	
Spells of terror or panic	0.32	1.02	
Feeling so restless you couldn’t sit still	0.61	1.07	
Feeling fearful	0.56	1.07	
Somatization (Index)	0.35	0.45	
Faintness or dizziness	0.18	0.42	
Pains in heart or chest	0.19	0.45	
Nausea or upset stomach	0.37	0.69	
Trouble getting your breath	0.36	0.79	
Numbness or tingling in parts of your body	0.52	0.91	
Feeling weak in parts of your body	0.50	0.81	
GSI (mean index)	0.53	0.51	0.888

Participants responded to the prompt: “How much were you bothered or distressed by the following problems in the past 7 days, including today?”

### Ryff’s psychological Well-being scale

The Ryff’s [30] psychological well-being scale covers six core dimensions of well-being and happiness: autonomy, environmental mastery, personal growth, positive relations with others, purpose in life, and self-acceptance. In this study, the shortened 18-item version [23] was used to evaluate participants’ overall eudaimonic well-being. Respondents rated their agreement with the 18 statements on a 7-point Likert scale ranging from 1 (strongly agree) to 7 (strongly disagree). This scale has been widely validated and applied across various populations [23, 30, 41, 42]. A German version used in previous research [43] was administered in this study. An eudaimonic well-being index was calculated as the average of the 18 items. Cronbach’s α value of 0.725 indicates good scale reliability [40].

**Table 3:** Ryff’s well-being scale

Item (1 = strongly disagree; 7 = strongly agree)	Mean	SD	Cronbach’s α
Autonomy	4.76	1.21	
I tend to be influenced by people with strong opinions^a^	5.19	1.82	
I have confidence in my own opinions, even if they are different from the way most other people think	4.46	1.81	
I judge myself by what I think is important, not by the values of what others think is important	4.63	1.69	
Environmental mastery	4.77	1.22	
In general, I feel I am in charge of the situation in which I live	4.80	1.60	
The demands of everyday life often get me down^a^	4.82	1.97	
I am good at managing the responsibilities of daily life	4.69	1.67	
Self-acceptance	4.04	1.11	
In many ways I feel disappointed about my achievements in life^a^	4.24	1.76	
When I look at the story of my life, I am pleased with how things have turned out so far	3.33	1.59	
I like most parts of my personality	4.56	1.66	
Personal growth	4.65	1.23	
I think it is important to have new experiences that challenge how I think about myself and the world	5.05	1.56	
For me, life has been a continuous process of learning, changing, and growth	4.65	1.70	
I gave up trying to make big improvements or changes in my life a long time ago^a^	5.18	1.73	
Positive relations	4.96	1.12	
Maintaining close relationships has been difficult and frustrating for me^a^	4.71	1.97	
I have not experienced many warm and trusting relationships with others^a^	4.25	1.91	
People would describe me as a giving person, willing to share my time with others	4.60	1.65	
Purpose in life	4.52	1.26	
I live life one day at a time and don’t really think about the future^a^	4.40	1.93	
I sometimes feel as if I’ve done all there is to do in life^a^	5.06	1.72	
Some people wander aimlessly through life, but I am not one of them	4.49	2.07	
Eudaimonic well-being (mean index)	4.62	0.79	0.725

Participants responded to the prompt: “Please indicate how much the following statements apply to you personally. Read each item carefully and rate the extent to which you agree with it.”; ^a^Item recoded into (1 = strongly agree; 7 strongly disagree)

### Positive and negative affect scale

The PANAS [31] measures positive and negative affect with ten items for each dimension on a five-point Likert scale (ranging from 1 = not at all to 5 = extremely). A validated German version of the PANAS [44] was used in the present study. An index was calculated for positive and for negative affect as the average of the ten items for each dimension. Cronbach’s α of the positive affect scale was 0.810 and 0.828 for the negative affect scale, indicating very good reliability [40].

**Table 4:** Positive and negative affect scale

Item (1 = not at all; 5 = extremely)	Mean	SD	Cronbach’s α
Positive affect (mean index)	2.95	0.71	0.810
Active	3.41	1.45	
Interested	3.44	1.03	
Enthusiastic	2.84	1.28	
Strong	3.21	1.33	
Inspired	2.00	1.14	
Proud	2.91	1.45	
Excited	2.62	1.37	
Alert	2.89	1.28	
Determined	2.93	1.24	
Attentive	3.30	1.10	
Negative affect (mean index)	2.12	0.73	0.828
Distressed	2.75	1.23	
Upset	2.59	1.29	
Guilty	2.35	1.41	
Scared	1.57	1.01	
Hostile	1.80	1.08	
Irritable	2.56	1.21	
Ashamed	1.62	1.02	
Nervous	2.01	1.23	
Jittery	2.27	1.31	
Afraid	1.70	1.18	

Participants responded to the prompt: “We would now like to know how you generally feel. The following words describe different feelings and emotions. Please read each word carefully and use the scale next to each word to indicate the extent to which you have experienced that feeling”

### Sample characteristics

To gain an overview of the sample characteristics, several demographic variables and sentence characteristics were assessed, including respondents’ age, height, and weight to calculate the body mass index (BMI), and the total sentence length as well as the prison sentence served until the time of study participation.

### Empirical analysis

All statistical analyses were conducted using IBM SPSS Statistics, Version 30. The empirical analysis consisted of two components. First, a two-step cluster analysis was conducted to address RQ1. This analysis was based on the following five behavioral cluster variables: (1) daily vigorous PA, (2) daily moderate PA, (3) daily walking PA, as well as daily sitting time, and (5) daily TV time. The number of clusters was determined by the algorithm based on the Schwarz’s Bayesian Criterion, which identified a three-cluster solution as optimal.

Second, to examine differences in respondent characteristics across these clusters, analyses of variances (ANOVAs) were performed. ANOVA is a commonly used method to test for group differences in means, and it is considered sufficiently robust [45]. ANOVAs were selected over ANCOVAs because key sociodemographic covariates (i.e., age, sentence length, and BMI) did not significantly differ across clusters (Table 5), and, thus, confounding was considered unlikely. Nevertheless, to assess the robustness of results, exploratory ANCOVAs were also conducted, adjusting for these covariates. The findings remained consistent and are reported in the supplementary materials (Supplementary Material 2). Furthermore, a Welch ANOVA was used for variables where the assumption of homogeneity of variances was violated (as indicated by a significant Levene’s test, *p* <.05). When an ANOVA revealed significant group differences, either Tukey’s HSD test or the Games-Howell post-hoc test was applied, depending on the assumption of variance homogeneity. Tukey’s HSD is recommended when group variances are equal, while the Games-Howell procedure is more appropriate when this assumption is violated, as it does not require equal variances or equal sample sizes [46].

**Table 5:** Cluster profiles based on daily activity, sedentary behavior, and TV time

	Cluster 1: High media consumption, low-intensity activity	Cluster 2: Highly active, low sedentary behavior	Cluster 3: Low active, low media time	F/Welch’s F
No. of rs.	35	52	41	
Cluster variables				
Daily vigorous PA	13.62^bc^	73.43^ac^	27.11^ab^	37.382***^w^
Daily moderate PA	35.18^b^	104.53^ac^	19.01^b^	84.600***^w^
Daily walking PA	91.10^c^	80.32^c^	11.70^ab^	42.900***^w^
Daily sitting time	9.18^bc^	3.98^a^	3.79^a^	58.981***
Daily TV time	5.94^bc^	2.27^a^	2.16^a^	28.540***^w^
Group characteristics				
Weekly MET minutes	3852.11^bc^	8894.532^ac^	2320.91^ab^	102.683***^w^
Life satisfaction	5.51	6.06	5.76	0.411
GSI	0.57	0.54	0.43	0.742
Eudaimonic well-being	4.62	4.80	4.51	1.702
Positive affect	2.71^b^	3.14^a^	3.07	3.671**
Negative affect	2.07	2.11	1.99	0.370
Age	21.23	22.15	21.61	2.198
BMI	26.46	24.78	25.80	1.800
Total prison sentence	25.57	27.44	25.56	0.202
Prison sentence served	9.18	9.92	10.02	0.038

Displayed are the mean values; *p <.10; **p <.05; ***p <.01; results of Tukey HSD post-hoc test for ANOVA or Games-Howell post-hoc test for Welch test: ^a^significantly different from cluster 1; ^b^significantly different from cluster 2; ^c^significantly different from cluster 3; ^w^Welch corrected F-value due to hetero variances

## Results

To gain an overview of the general sample structure, Table 1 provides the descriptive statistics. On Average, Prisoners reported more than two-and-a-half hours of daily PA, which surpasses the World Health Organization’s recommendation of 21–43 min of moderate or 11–22 min of vigorous activity per day [9]. They averaged about 42.24 min of vigorous intensity activity, a little less than one hour (58.18 min) of moderate intensity activity, and 61.29 min spent walking. To complete the picture, prisoners spent more than five hours per day sitting, with approximately 60% of that time (3.24 h), spent watching TV. Their overall weekly PA level is equivalent to 5410.13 MET minutes.

Regarding PWB indicators, the mean life satisfaction score was 5.81 on a scale from 0 to 10. The average score on the GSI, indicating psychological distress, was 0.52 (scale 0–4). Average eudaimonic well-being was rated at 4.66 on a 7-point Likert scale. Positive affect was reported with a mean of 3.00, while negative affect averaged 2.06 (scale 1–5).

At the time of data collection, prisoners were on average 21.73 years old. The average BMI of prisoners was 25.57, which, following the BMI classifications, is slightly overweight [47]. The average sentence length was about two years and two months, of which more than a third (9.80 months) was already served.

Next, to further address RQ1, which aimed to identify distinct behavioral profiles among prisoners, a two-step cluster analysis was performed based on the five key indicators of physical activity and sedentary behavior. This analysis yielded three distinct clusters that are presented in Table 5 and were labelled as follows: Cluster 1 (High media consumption, low-intensity activity), Cluster 2 (Highly active, low sedentary behavior), and Cluster 3 (Low active, low media time). With 35, 52, and 41 participants respectively, the clusters are relatively balanced in size, with Cluster 2 being the largest. The cluster labels reflect systematic differences in activity and sedentary profiles. Cluster 1 exhibited the highest TV viewing and sitting time combined with low-to-moderate activity levels, supporting the label “High media consumption, low-intensity activity”. Participants in Cluster 2 spent the most time engaged in vigorous and moderate PA, and spent the least amount of time being sedentary, which justified their classification as “Highly active, low sedentary behavior”. Those in Cluster 3 showed low levels of PA across all intensities and were the least sedentary; hence, they were labelled “Low active, low media time”.

In terms of PA, participants in Cluster 2 reported the highest levels of daily vigorous PA (73.43 min), followed by Cluster 3 (27.11 min) and Cluster 1 (13.62 min). Similarly, moderate activity levels were greatest in Cluster 2 (104.53 min), with notably lower levels in Cluster 1 (35.18 min) and Cluster 3 (19.01 min). Walking activity was highest in Cluster 1 (91.10 min), slightly lower in Cluster 2 (80.32 min), and considerably lower in Cluster 3 (11.70 min).

Regarding sedentary behavior, Cluster 1 reported the highest daily sitting time (9.18 h) and TV viewing time (5.94 h), while Clusters 2 and 3 both reported significantly lower sedentary behavior (around 4 h sitting and 2.2 h of TV time daily). Post-hoc tests revealed that these differences across clusters were statistically significant.

Looking at the ANOVAs, statistically significant differences were found for two group characteristics. First, Weekly MET minutes differed significantly across clusters (*Welch’s F* = 102.683, *p* <.001). Prisoners in the “Highly active, low sedentary behavior” cluster demonstrated the highest weekly energy expenditure (M = 8,894.53), followed by the “High media consumption, low-intensity activity” cluster (M = 3,852.11), with the lowest levels found in the “Low active, low media time” cluster (M = 2,320.91). Games- Howell post-hoc tests confirmed significant differences between all three clusters (*p* <.05 for all comparisons).

Second, regarding positive affect, the analysis revealed a significant overall difference among clusters (*F* = 3.671, *p* =.028). Prisoners in the “Highly active, low sedentary behavior” cluster reported significantly higher positive affect (M = 3.14) compared to those in the “High media consumption, low-intensity activity” cluster (M = 2.71). The “Low active, low media time” cluster (M = 3.07) did not differ significantly from the others. Tukey HSD post-hoc comparisons indicated a significant difference between Clusters 1 and 2 (*p* =.027). No statistically significant differences were found across clusters for the remaining PWB indicators, including life satisfaction (*F* = 0.411, *p* =.664), psychological distress (GSI; *F* = 0.742, *p* =.478), eudaimonic well-being (*F* = 1.702, *p* =.186), and negative affect (*F* = 0.370, *p* =.691).

### Discussion and conclusions

The aim of this study was to identify distinct behavioral profiles of PA and sedentary behavior among prisoners in an open correctional facility (RQ1) and to explore whether these behavioral profiles differ in terms of PWB (RQ2). Using five activity-related indicators (vigorous, moderate, and walking PA, sitting time, and TV viewing), the cluster analysis revealed three subgroups.

The first group, “High media consumption, low-intensity activity,” displayed low engagement in all forms of physical activity (PA), including walking, moderate, and vigorous intensity, and reported the highest levels of sedentary behavior, with over three hours of daily TV viewing. The second cluster, “Highly active, low sedentary behavior,” represented the healthiest profile, combining high values in all PA intensities with the lowest sedentary time. In contrast, the third group, “Low active, low media time,” reported low values for both physical activity and screen-based behavior, reflecting a more passive or withdrawn lifestyle. These findings demonstrate that even within a regulated correctional environment, behavioral heterogeneity exists in terms of how inmates organize their movement and media routines. Such patterns would remain hidden in aggregated analyses, highlighting the value of a behavioral segmentation-based approach.

When benchmarked against the World Health Organization’s recommendations for adult physical activity (150 min of moderate or 75 min of vigorous PA per week, or an equivalent of 600 MET minutes per week) [9], the overall sample showed high levels of reported movement. The average weekly MET minutes amounted to 5,410, with Cluster 2 exceeding 8,800 MET minutes. While this finding suggests that most inmates in our sample surpass minimum PA recommendations, the exceptionally high values raise the possibility of overreporting, a known limitation of self-report instruments like the IPAQ [48]. Given the reliance on retrospective recall and subjective estimates, participants may have unintentionally exaggerated their activity durations. At the same time, the specific circumstances of daily prison life should be considered: These include walking between housing or activity units, attending roll calls, participating in assigned duties such as gardening, bricklaying, kitchen or maintenance work, and commuting, often by foot or bicycle, and at least to the nearest public transport stop, as motorized transport is mostly unavailable, to external education, training, or work programs. Such practical forms of activity contribute to total PA levels and may help explain the elevated values observed in this population. This aligns with earlier findings by Mannocci et al. [17, 18], who reported even higher values among Italian prisoners (on average, approximately 6,900 or 9,200 MET-min/week) [17, 18]. These parallels support the credibility of the reported activity levels in our sample, especially in environments where PA is embedded in daily routines. However, prisoners in Cluster 3 fell slightly below the recommended 150 min of moderate-intensity physical activity per week, reporting an average of 133 min. Nonetheless, they appeared to compensate for this shortfall through higher engagement in vigorous-intensity activity, which contributed to meeting the overall MET-based recommendations. These results indicate that most incarcerated individuals in this sample meet and even exceed basic health-enhancing physical activity recommendations.

This does not necessarily contradict the framing of open prisons as environments that allow for greater self-determination. However, PA in prison, as outlined above, is often embedded in institutional routines rather than reflecting intrinsic motivation or health-oriented intentions. This distinction is critical, as research consistently shows that the benefits of physical activity for psychological and physical well-being depend not only on its intensity or duration but also on whether it is undertaken voluntarily [49, 50]. Overall, this suggests that both voluntary and non-voluntary forms of PA likely coexist. While inmates may have the autonomy to engage in discretionary leisure-time activity (e.g., sports or exercise), a substantial proportion of their movement may still arise from routine tasks or required practical tasks. Given the nature of the IPAQ-short form, which does not distinguish between these sources of activity [32], the high PA levels observed in this study should be interpreted as total movement, not necessarily intentional or autonomy-driven behavior.

Wicker and Frick [51], analyzing a large European sample of the general population found that respondents, considering their weekly PA, only spent a little less than 40 min walking, 143 min of moderate intensity PA and 106 min in vigorous PA, which is substantially lower than observed among the prisoners in our sample. This finding contrasts assumptions that prison environments decrease PA and enhance sedentary behavior. Here, the unique structure of PA in correctional settings, where high activity levels may result from situational constraints rather than leisure-time preferences, comes into play. Nonetheless, the physical health benefits remain relevant, particularly when considering long-term risks of sedentary behavior or physical inactivity, even if the psychological advantages are less consistent under non-volitional conditions.

Despite these relatively high PA levels, the average BMI in this sample remained in the slightly overweight range (mean: 26.3), which aligns with previous observations from Mannocci et al. in Italian prison populations [17, 18]. This finding suggests that physical activity alone, especially when it emerges from daily routines rather than targeted interventions, may be insufficient to counteract other risk factors such as dietary habits, stress, or environmental constraints. While Dransmann et al. [52] reported a significant BMI reduction following a 6-week structured high-intensity interval training program, their study involved a clearly defined training protocol and close supervision, conditions that differ fundamentally from the unstructured and routine-based activity patterns assessed here. Thus, activity alone, especially when not paired with physical activity or dietary interventions or health education, may be insufficient to counteract other behavioral or environmental risk factors.

In terms of sedentary behavior, inmates reported an average of over five hours of sitting per day, with roughly three of those hours attributed to TV viewing. While high screen time is generally considered a risk factor for physical and mental health [23, 24], its interpretation in the prison context is more complex. TV represents one of the few accessible leisure options and can offer routine, distraction, or even a sense of autonomy in an otherwise highly regulated environment [53, 54]. Accordingly, interventions aiming to reduce screen time must consider its psychological role as a coping mechanism and perceived form of self-determination. Together, these cluster-specific patterns provide nuanced insights into the behavioral health landscape of open prisons. Compared to existing literature, the present study extends prior research by Mannocci et al. [17, 18] through its differentiation of PA intensities, its integration of media-based sedentary behavior, and its empirical identification of naturally occurring behavioral profiles.

The findings underscore that it is not PA in isolation, but the co-occurrence of physical activity, sedentary behavior, and media use that defines distinct health-relevant behavioral profiles in prison. These patterns are relevant for broader dimensions of general health. Sedentary behavior is a well-established risk factor for numerous chronic conditions, including cardiovascular disease, type 2 diabetes, and metabolic syndrome [14, 15]. Thus, behavior patterns identified here may signal long-term health trajectories beyond the prison context.

Regarding PWB (RQ2), positive affect was the only psychological health measure that significantly differed between clusters. Specifically, the “Highly active, low sedentary” group reported higher positive affect than the “High media consumption, low-intensity” group. This finding is consistent with literature indicating that PA is linked to enhanced mood and emotional stability, both in general [6, 55] and prison populations [56]. However, no statistically significant differences were found for the other well-being components: life satisfaction, eudaimonic well-being, psychological distress (GSI), and negative affect. These non-findings suggest that variations in everyday behavioral patterns may not be sufficient to meaningfully impact more stable or multifaceted dimensions of well-being. This may reflect the limited psychological impact of PA when it is not voluntarily chosen or done as a leisure time activity [49, 50]. In the prison context, much of the recorded activity may stem from obligations, such as commuting by foot or public transport to mandatory school or work programs. Such forms of movement, while physically activating, are unlikely to elicit psychological benefits, as they are routine-driven, externally imposed, and often perceived as chores rather than opportunities for autonomy or enjoyment. This aligns with prior research by Wicker [57], who found that travel to regular training sessions was positively associated with subjective well-being, whereas travel for competitive purposes had no such effect on well-being. Similarly, Rasciute and Downward [58] found that active travel in the form of cycling, while beneficial for physical health, may not enhance or even decrease well-being.

The findings of the present study partially contrast with prior research based on structured programs, such as yoga, strength training, or team sports [10–13, 59], which have shown more robust effects across various well-being indicators. However, earlier studies typically investigated short-term intervention effects [10–13, 59], whereas our study captured habitual, unstructured activity patterns. Contextual limitations of prison life, such as reduced autonomy and routine-driven behavior, may reduce the psychological relevance of PA when it is not chosen freely.

Sample characteristics may also play a role. The current sample was relatively young and already physically active, potentially limiting variance in both behavioral and psychological indicators. Furthermore, some well-being domains, particularly eudaimonic aspects such as autonomy and purpose, may be more stable and less responsive to behavioral variation in restrictive environments.

Comparisons with the resident population offer additional context. Life satisfaction among prisoners was lower than in the general German population (5.81 vs. 6.72) [60], while positive and negative affect were only slightly less favorable (3.00 vs. 3.19; 2.06 vs. 1.85) [61]. Interestingly, GSI scores (0.52) were lower than general, non-incarcerated population values (1.02) [62], and eudaimonic well-being scores (4.66 on a 7-point scale) were relatively comparable to German adults’ averages (3.57 on a 6-point scale) [63]. These patterns suggest that some deeper components of well-being, like meaning or self-acceptance, may be preserved even in prison environments.

By empirically identifying three distinct subgroups, this study provides a foundation for more differentiated health promotion strategies in correctional settings. A one-size-fits-all approach is unlikely to be effective given the diversity in movement and media routines. Instead, profile-based interventions can better address individual needs and starting points. For instance, the “High media consumption, low-intensity activity” group may face elevated risk not only for psychological strain but also for physical health deterioration due to insufficient movement and prolonged sitting. Tailored programs should aim to increase light-intensity activity and reduce passive behavior without undermining the role of TV as a coping mechanism and source of perceived autonomy. Supporting this group in building healthier routines could yield long-term benefits for both mental and physical health.

The “Highly active, low sedentary” group may already maintain beneficial patterns. Sustaining this behavior through autonomy-supportive programs, variety in physical activities, and peer-based engagement may help to preserve both emotional resilience and physical fitness. The “Low active, low media time” group represents a particularly vulnerable segment. Their limited engagement in both domains may reflect passivity or depressive symptoms. This group may benefit most from low-threshold activation programs and psychosocial support, especially to strengthen autonomy and purpose, key components of eudaimonic well-being.

Ultimately, these findings highlight the need for differentiated health promotion strategies within prisons. Behavioral profiles offer a practical basis for tailoring programs, whether by activating sedentary inmates, supporting active routines, or enhancing autonomy through flexible, meaningful activities. Promoting balanced movement and screen habits could support both prisoner well-being and long-term reintegration success. Notably, improving physical and mental health in prison is not only a matter of institutional well-being but also of societal relevance. Incarcerated individuals are likely to reenter society, where poor health may hinder employment opportunities and burden public health systems [64, 65]. A healthier, more active prison population may thus contribute to labor market reintegration and reduce long-term healthcare costs [64, 65]. These findings support efforts to design holistic and sustainable health strategies that benefit both the individual and society.

Several limitations should be acknowledged. First, the cross-sectional design limits causal interpretations. Future studies should consider longitudinal approaches to assess how behavioral patterns and well-being develop over time. Second, all data were self-reported, which may be subject to bias, including potential over- or underreporting of physical activity levels, particularly in correctional settings where trust and privacy are delicate issues [66, 67]. While implausible values were removed following the IPAQ scoring protocol, future studies should consider objective measures (e.g., pedometers) and more systematic sampling. Moreover, since the IPAQ does not differentiate between voluntary and compulsory activity, future studies should consider tools that capture the motivational context of PA, as this may moderate its effects on well-being. Third, the sample was limited to male inmates in a single German open prison. Results may not generalize to other settings, including closed facilities [59], female populations [68], or older prisoners [69]. Finally, future research should explore the phenomenon of high physical activity levels in open prisons more systematically. Such elevated values may not only reflect methodological bias (e.g., self-report, recall), but also structural features of the prison environment (e.g., routine-based or transport-related movement) that unintentionally promote physical activity. Investigating these contextual drivers in comparison to closed settings could help identify environmental spillover effects and inform prison health policy.

## Supplementary Information

Below is the link to the electronic supplementary material.

Supplementary Material 1

## Data Availability

The datasets used and/or analyzed during the current study are available from the corresponding author on reasonable request.

## Abbreviations

ANOVA: Analysis of variance
BMI: Body mass index
BSI-18: Brief symptom inventory
BSI-18: Brief symptom inventory
GSI: Global severity index
IPAQ: International physical activity questionnaire
MET: Metabolic equivalent of task
PA: Physical activity
PANAS: Positive and negative affect scale
PWB: Psychological well-being
RQ: Research question
TV: Television
WHO: World Health Organization
