# Supplementary material for: Physical activity and sedentary behavior profiles among prisoners in an open prison: differences in psychological well-being?
Source: BMC Public Health. 2025 Sep 1;25:2999. doi: 10.1186/s12889-025-24345-0 (PMC12400634; doi:10.1186/s12889-025-24345-0)
Supplement: Supplementary file 1 — Supplementary Material 1 [file 12889_2025_24345_MOESM1_ESM.docx]

**Supplementary Material 1 - English Version of Study Questionnaire**

Dear Sir or Madam,

Thank you for your interest in participating in this survey.

**Purpose of the Study**

This survey is part of a research project by Mr. Christian Kraft, focusing on the topic „The relationship between physical activity and psychological well-being among prisoners“.

**Procedure and Content of the Survey**

Participation in the survey will take approximately 25 to 35 minutes. It is aimed at female and male prisoners in correctional facilities. The minimum age for participation is 18 years.

You will receive the questionnaire in an envelope and complete it privately in your cell. After completion, please return the sealed envelope. This procedure ensures that your responses cannot be linked to you personally.

The questionnaire covers your physical activity, the amount of time you spend sitting during the day, and your psychological well-being. In addition, it collects information about your age, gender, family situation, and the duration of your sentence.

**Data Protection, Data Access, and Duration of Data Storage**

The data will be collected **anonymously**. It will not be possible to identify you or any other individuals based on your responses.

The anonymized data will be stored on a password-protected server, and, according to the University of Bielefeld's retention guidelines, will be kept for 10 years to allow use for future research projects.

**Data Publication**Your data will be used **exclusively for scientific purposes** and treated with strict confidentiality. Presentations to third parties (e.g., in scientific publications or lectures) will not allow any conclusions to be drawn about individual participants due to the anonymous nature of data collection.

**Risks** There are no expected negative consequences or particular burdens associated with participation in the survey.

The questions about mental health in the questionnaire do not replace professional psychological diagnostics. They are intended merely to provide indications of psychological well-being.

In case of psychological problems, please contact the psychological services.

**Voluntary Participation** Participation in the survey is voluntary. You can discontinue the survey at any time without providing reasons and without facing any disadvantages.

**Contact Information** If you have any questions, please contact:

Christian Kraft (christian.kraft@uni-bielefeld.de) or +49 521-106-2009

**Consent**

I have read and understood the information regarding participation in the survey. I feel sufficiently informed and had the opportunity to ask questions.

I am aware that participation in the survey is voluntary and that refusing consent will not result in any disadvantages. I can withdraw my consent at any time by discontinuing the survey. No justification is required for withdrawal. After completing the questionnaire, it will no longer be possible to withdraw consent, as the anonymized data can no longer be assigned to individual participants.

By checking the box, you confirm your consent to participate in the study.

I hereby confirm my participation in the study.

Here is a list of problems people sometimes have. Read each statement and indicate: How much that problem has distressed or bothered you during the past 7 days, including today.

Please make sure to answer each question.

| During the past 7 days, how much were you distressed by… | | | | | |
| --- | --- | --- | --- | --- | --- |
|  | not at all | a little bit | moderately | quite a bit | extremely |
| Faintness or dizziness | □ | □ | □ | □ | □ |
| Feeling of no interest in things | □ | □ | □ | □ | □ |
| Nervousness or shakiness | □ | □ | □ | □ | □ |
| Pains in heart or chest | □ | □ | □ | □ | □ |
| Feeling lonely | □ | □ | □ | □ | □ |
| Feeling tense or keyed up | □ | □ | □ | □ | □ |
| Nausea or upset stomach | □ | □ | □ | □ | □ |
| Feeling blue | □ | □ | □ | □ | □ |
| Suddenly scared for no reason | □ | □ | □ | □ | □ |
| Trouble getting your breath | □ | □ | □ | □ | □ |
| Feelings of worthlessness | □ | □ | □ | □ | □ |
| Spells of terror or panic | □ | □ | □ | □ | □ |
| Numbness or tingling in parts of your body | □ | □ | □ | □ | □ |
| Feeling hopeless about the future | □ | □ | □ | □ | □ |
| Feeling so restless you couldn’t sit still | □ | □ | □ | □ | □ |
| Feeling weak in parts of your body | □ | □ | □ | □ | □ |
| Thoughts of ending your life | □ | □ | □ | □ | □ |
| Feeling fearful | □ | □ | □ | □ | □ |

This scale consists of a number of words that describe different feelings and emotions. Read each item and then mark the appropriate answer in the space next to that word. Indicate to what extent you feel this way in general. Use the following scale to record your answers.

|  | not at all | a little bit | moderately | quite a bit | extremely |
| --- | --- | --- | --- | --- | --- |
| Interested | □ | □ | □ | □ | □ |
| Distressed | □ | □ | □ | □ | □ |
| Excited | □ | □ | □ | □ | □ |
| Upset | □ | □ | □ | □ | □ |
| Strong | □ | □ | □ | □ | □ |
| Guilty | □ | □ | □ | □ | □ |
| Scared | □ | □ | □ | □ | □ |
| Hostile | □ | □ | □ | □ | □ |
| Enthusiastic | □ | □ | □ | □ | □ |
| Proud | □ | □ | □ | □ | □ |
| Irritable | □ | □ | □ | □ | □ |
| Alert | □ | □ | □ | □ | □ |
| Ashamed | □ | □ | □ | □ | □ |
| Inspired | □ | □ | □ | □ | □ |
| Nervous | □ | □ | □ | □ | □ |
| Determined | □ | □ | □ | □ | □ |
| Attentive | □ | □ | □ | □ | □ |
| Jittery | □ | □ | □ | □ | □ |
| Active | □ | □ | □ | □ | □ |
| Afraid | □ | □ | □ | □ | □ |

In general, how satisfied are you with your life?

| Very dissatisfied Very satisfied | | | | | | | | | | |
| --- | --- | --- | --- | --- | --- | --- | --- | --- | --- | --- |
| □ | □ | □ | □ | □ | □ | □ | □ | □ | □ | □ |
| 0 | 1 | 2 | 3 | 4 | 5 | 6 | 7 | 8 | 9 | 10 |

Please indicate how much the following statements apply to you personally.

|  | strongly disagree | somewhat disagree | a little disagree | neither agree nor disagree | a little agree | somewhat agree | strongly agree |
| --- | --- | --- | --- | --- | --- | --- | --- |
| I tend to be influenced by people with strong opinions. | □ | □ | □ | □ | □ | □ | □ |
| In general, I feel I am in charge of the situation in which I live. | □ | □ | □ | □ | □ | □ | □ |
| In many ways I feel disappointed about my achievements in life. | □ | □ | □ | □ | □ | □ | □ |
| I think it is important to have new experiences that challenge how I think about myself and the world. | □ | □ | □ | □ | □ | □ | □ |
| Maintaining close relationships has been difficult and frustrating for me. | □ | □ | □ | □ | □ | □ | □ |
| I live life one day at a time and don’t really think about the future. | □ | □ | □ | □ | □ | □ | □ |
| When I look at the story of my life, I am pleased with how things have turned out so far. | □ | □ | □ | □ | □ | □ | □ |
| I sometimes feel as if I’ve done all there is to do in life. | □ | □ | □ | □ | □ | □ | □ |
| I have confidence in my own opinions, even if they are different from the way most other people think. | □ | □ | □ | □ | □ | □ | □ |
| I have not experienced many warm and trusting relationships with others. | □ | □ | □ | □ | □ | □ | □ |
| The demands of everyday life often get me down. | □ | □ | □ | □ | □ | □ | □ |
| For me, life has been a continuous process of learning, changing, and growth. | □ | □ | □ | □ | □ | □ | □ |
| People would describe me as a giving person, willing to share my time with others. | □ | □ | □ | □ | □ | □ | □ |
| I gave up trying to make big improvements or changes in my life a long time ago. | □ | □ | □ | □ | □ | □ | □ |
| Some people wander aimlessly through life, but I am not one of them. | □ | □ | □ | □ | □ | □ | □ |
| I like most parts of my personality. | □ | □ | □ | □ | □ | □ | □ |
| I judge myself by what I think is important, not by the values of what others think is important. | □ | □ | □ | □ | □ | □ | □ |
| I am good at managing the responsibilities of daily life. | □ | □ | □ | □ | □ | □ | □ |

We are interested in finding out about the types of physical activities that people do as part of their everyday lives. The questions will assess the time you spent being physically active in the **last 7 days**. Please answer each question even if you do not consider yourself to be an active person. Please think about the activities you do at work, in prison, to get from place to place, and in your spare time for recreation, exercise, or sport.

1. Think about all the **vigorous** activities that you did in the **last 7 days**. **Vigorous** physical activities refer to activities that take hard physical effort and make you breathe much harder than normal. Think only about those physical activities that you did for at least 10 minutes at a time. During the **last 7 days**, on how many days did you do **vigorous** physical activities like running/jogging, fast cycling (stationary bike), playing basketball or soccer, or carrying heavy loads (construction work)?

| **days per week ❑ No vigorous physical activities (skip to question 3)** |
| --- |

1. How much time did you usually spend doing vigorous physical activities on one of those days?

**hours and minutes per day**

1. Think about all the **moderate** activities that you did in the **last 7 days**. **Moderate** activities refer to activities that take moderate physical effort and make you breathe somewhat harder than normal. Think only about those physical activities that you did for at least 10 minutes at a time. During the **last 7 days**, on how many days did you do **moderate** physical activities like carrying light loads (gardening, manual work), strength training, playing table tennis, or cycling (stationary bike) at a regular pace? Do not include walking.

**days per week ❑ No moderate physical activity (skip to question 5)**

1. How much time did you usually spend doing moderate physical activities on one of those days?

**hours and minutes per day**

1. Think about the time you spent **walking** in the **last 7 days**. This includes walking at work and in prison, walking to travel from place to place, and any other walking that you have done solely for recreation, sport, exercise, or leisure. During the **last 7 days**, on how many days did you **walk** for at least 10 minutes at a time?

**days per week ❑ No walking (skip to question 7)**

1. How much time did you usually spend **walking** on one of those days?

**hours and minutes per day**

1. The last question is about the time you spent **sitting** on weekdays during the **last 7 days**. Include time spent at work, at home, while doing course work, and during leisure time. This may include time spent sitting during visits, playing games, sitting in front of the television, sitting or lying on the bed while engaging in various activities (reading, writing), and also sitting during meals. During the last 7 days, how much time did you spend sitting on a week day?

**hours and minutes per day**

1. Out of that time, how many hours did you spend **watching television**?

**hours and minutes per day**

Please indicate how much you agree with the following statements.

|  | strongly disagree | disagree | neither agree nor disagree | agree | strongly agree |
| --- | --- | --- | --- | --- | --- |
| The previously described week closely resembles other weeks during my time in prison. | □ | □ | □ | □ | □ |
| The previously described week closely resembles my everyday life **before** prison | □ | □ | □ | □ | □ |

Please indicate how much you agree with the following statement.

| Before incarceration, I was.… | | | | |
| --- | --- | --- | --- | --- |
| …much less physically active | …somewhat less physically active | …about the same amount  physically active | …somewhat more physically active | … much more physically active |
| □ | □ | □ | □ | □ |

**Demographic questions:**

**What is your gender?**

□ male □ female □ non-binary

**How old are you?** years

**How tall are you?** cm

**How much do you weigh?** kg

**What is your marital status?**

□ single
□ divorced
□ widowed

□ married

**Do you or one of your parents hold a non-German citizenship?**

□ Yes □ No

**Please indicate approximately how many visits you receive per month.**

**Please indicate approximately how many letters you exchange with your family or close friends per month.**

**What is your highest level of education?**
□ still in school

□ no degree/dropped out of school

□ special needs school
□ certificate of secondary education
□ general certificate of secondary education

□ university of applied sciences entrance qualification
□ general university entrance qualification,

□ other (please specify):

**What is the total length of your prison sentence?**

years months

**How long have you already been in prison? (Period from the start of imprisonment until now)**

years months

**Supplementary Material 2 – Table of ANCOVA**

|  | Cluster 1: High media consumption, low-intensity activity | Cluster 2: Highly active, low sedentary behavior | Cluster 3: Low active, low media time | *F* |
| --- | --- | --- | --- | --- |
| No. of rs. | 35 | 52 | 41 |  |
| Cluster variables |  |  |  |  |
| Daily vigorous PA | 13.62^b^ | 73.43^ac^ | 27.11^b^ | 37.812*** |
| Daily moderate PA | 35.18^b^ | 104.53^ac^ | 19.01^b^ | 57.183*** |
| Daily walking PA | 91.10^c^ | 80.32^c^ | 11.70^ab^ | 22.986*** |
| Daily sitting time | 9.18^bc^ | 3.98^a^ | 3.79^a^ | 55.450*** |
| Daily TV time | 5.94^bc^ | 2.27^a^ | 2.16^a^ | 42.394*** |
| Group characteristics |  |  |  |  |
| Weekly MET minutes | 3852.11^bc^ | 8894.532^ac^ | 2320.91^ab^ | 82.927*** |
| Life satisfaction | 5.51 | 6.06 | 5.76 | 0.129 |
| GSI | 0.57 | 0.54 | 0.43 | 0.887 |
| Eudaimonic well-being | 4.62 | 4.80 | 4.51 | 0.906 |
| Positive affect | 2.71^b^ | 3.14^a^ | 3.07 | 2.854* |
| Negative affect | 2.07 | 2.11 | 1.99 | 0.451 |

Note. Displayed are the mean values; **p* < 0.10; ***p* < 0.05; ****p* < 0.01; results of Bonferroni post-hoc test for ANCOV: ^a^significantly different from cluster 1; ^b^significantly different from cluster 2; ^c^significantly different from cluster 3; ^w^Welch corrected F-value due to hetero variances; controlled for covariates: age, BMI, and total prison sentence.
